# Emerging Role of Nicotinamide Riboside in Health and Diseases

**DOI:** 10.3390/nu14193889

**Published:** 2022-09-20

**Authors:** Chiranjeev Sharma, Dickson Donu, Yana Cen

**Affiliations:** 1 Department of Medicinal Chemistry, Virginia Commonwealth University, Richmond, VA 23219, USA; 2 Institute for Structural Biology, Drug Discovery and Development, Virginia Commonwealth University, Richmond, VA 23219, USA

**Keywords:** vitamin B3, nicotinamide riboside, health, COVID-19, dietary supplements

## Abstract

Among all the NAD^+^ precursors, nicotinamide riboside (NR) has gained the most attention as a potent NAD^+^-enhancement agent. This recently discovered vitamin, B3, has demonstrated excellent safety and efficacy profiles and is orally bioavailable in humans. Boosting intracellular NAD^+^ concentrations using NR has been shown to provide protective effects against a broad spectrum of pathological conditions, such as neurodegenerative diseases, diabetes, and hearing loss. In this review, an integrated overview of NR research will be presented. The role NR plays in the NAD^+^ biosynthetic pathway will be introduced, followed by a discussion on the synthesis of NR using chemical and enzymatic approaches. NR’s effects on regulating normal physiology and pathophysiology will also be presented, focusing on the studies published in the last five years.

## 1. Introduction

Vitamins are a group of structurally diversified, small organic molecules that are essential for almost all forms of life. Although often required in small amounts and designated as “micronutrients”, vitamins have proven critical for maintaining normal physiology. Their absence or deficiency is known to cause disorders or diseases such as anemia [1], beriberi [2], pellagra [3], scurvy [4], night blindness [5], and blood coagulation disorders [6]. The study of vitamins has advanced significantly during the last century, being recognized by the Nobel Prize Committee in the form of numerous awards for vitamin-related research since 1928 [7]. All the research efforts have also resulted in the development of dietary recommendations and vitamin supplementation for disease prevention and treatment [8,9,10]. The human nutrition market was worth 252.38 billion USD in 2020 and is expected to expand during the next decade [11]. This growth can be attributed to increased health awareness, as well as surging demand for additional protection from the devastating COVID-19 pandemic [12,13]. 

This review focuses on a newly discovered form of vitamin B3, nicotinamide riboside (NR). Initially, NR was shown to increase intracellular NAD^+^ concentrations and to extend the life span without calorie restriction (CR) in yeast [14]. Subsequent studies established that NR is a potent NAD^+^ booster [15,16,17]. Together with two other NAD^+^ precursors, nicotinamide (NAM) and nicotinic acid (NA), NR belongs to the vitamin B3 family [14]. The literature is replete with the beneficial effects of NR-mediated NAD^+^ elevation in a broad spectrum of diseases, including neurodegenerative diseases [18,19], metabolic disorders [20,21], and cardiac fibrosis [22]. Notably, SARS-CoV-2 infection disrupts NAD^+^ homeostasis by depleting cellular NAD^+^ contents and upregulating poly(ADP-ribose) polymerases (PARPs), the NAD^+^-utilizing enzymes [23,24]. Reduced NAD^+^ level has been shown to promote inflammation and increase cellular injury [25,26]. Consequently, NAD^+^-depletion may be held partially responsible for the higher mortality rate in patients with pre-existing medical conditions, such as respiratory diseases, cardiovascular diseases, and diabetes. PARP inhibitors have been suggested as potential therapeutics for COVID-19 by blocking virus proliferation, preventing immune cell hyperactivation, and reducing the levels of circulating cytokines [27]. Interestingly, restoring NAD^+^ concentration using precursors such as NR is also under intense investigation to attenuate COVID-19-induced complications [28]. 

In this review, our effort is directed at discussing the distinct NAD^+^ biosynthetic pathways; the synthesis of NR; the role NR plays in health promotion; and disease prevention. The therapeutic potential of NR in treating COVID-19 is discussed separately towards the end. Several databases, including SciFinder, PubMed, Google Scholar, and Researchgate, were researched for peer-reviewed articles on the subject matter. Special attention is given to the publications after 2018 for NR in health and diseases. Publications prior to that have also been included to establish the foundation knowledge of NR. 

## 2. NR and NAD^+^ Biosynthesis

**Figure 1:**
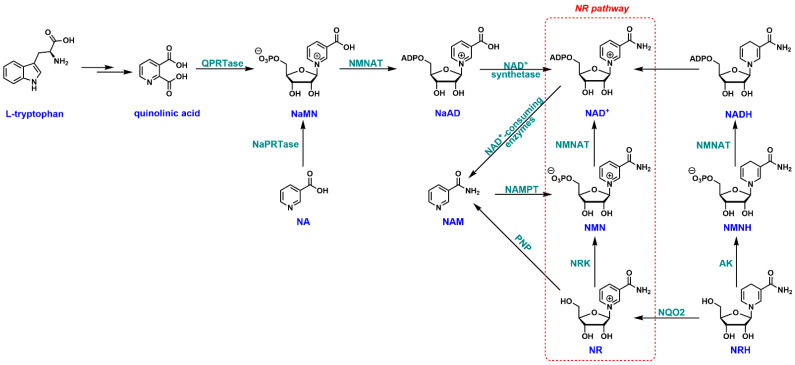
NAD^+^ biosynthetic pathways in mammalian cells. NA: nicotinic acid; NAM: nicotinamide; NR: nicotinamide riboside; NMN: nicotinamide mononucleotide; NaMN: nicotinic acid mononucleotide; NaAD: nicotinic acid adenine dinucleotide; NRH: reduced nicotinamide riboside; NMNH: reduced nicotinamide mononucleotide; QPRTase: quinolinate phosphoribosyltransferase; NMNAT: nicotinamide mononucelotide adenylyltransferase; NaPRTase: nicotinic acid phosphoribosyl transferase; NAMPT: nicotinamide phosphoribosyl transferase; NRK: nicotinamide riboside kinase; PNP: purine nucleoside phosphorylase; AK: adenosine kinase; NQO2: NRH: quinone oxidoreductase 2.

In mammals, an intricate NAD^+^ biosynthetic network has been established, including the de novo, salvage, NR, and dihydronicotinamide riboside (NRH) pathways (Figure 1) [29,30]. *L*-Tryptophan is the starting point of the de novo pathway [31]. It is enzymatically transformed through several steps to quinolinic acid, an immediate precursor of nicotinic acid mononucleotide (NaMN). Nicotinamide mononucleotide adenylyltransferase (NMNAT)-catalyzed adenylation converts NaMN to nicotinic acid adenine dinucleotide (NaAD), which can be further amidated to NAD^+^ by NAD^+^ synthetase. Intracellular NAD^+^ can be degraded to NAM by a class of enzymes called “NAD^+^-consuming enzymes”, such as sirtuins, poly(ADP-ribose) polymerase (PARP), and CD38 [32,33]. The salvage pathway is known to recycle NAM by nicotinamide phosphoribosyltransferase (NAMPT) to nicotinamide mononucleotide (NMN) [34]. NMN can be fully incorporated into NAD^+^ by the action of NMNAT. Another branch of the salvage pathway starts with NA. It is recycled by nicotinate phosphoribosyltransferase (NaPRTase) to NaMN through the Preiss–Handler pathway [35]. NaMN thus serves as a common intermediate for NA salvage and de novo NAD^+^ biosynthesis. NR is a naturally occurring metabolite initially found in milk [36]. It can be directly phosphorylated by NR kinases (NRK1/NRK2) to NMN [15,37,38], and ultimately to NAD^+^. This pathway has been extensively studied because NR is considered a potent NAD^+^ precursor [38,39,40], and boosting intracellular NAD^+^ content has been suggested as a potential anti-aging strategy [41,42,43]. Additionally, gut microbiota can also convert the dietary NR into various NAD^+^ precursors such as NAM, NA, and nicotinic acid riboside (NAR) in the colonic lumen and boost NAD^+^ biosynthesis [44]. NR is water-soluble and cell-permeable with no apparent toxicity [16]. Unlike other NAD^+^ precursors, such as NAM or NA, NR is not associated with any severe side effects [15,45]. All of these features render NR an ideal candidate as a therapeutic agent for NAD^+^ restoration. NRH is the “new kid on the block” as an NAD^+^ precursor. It has been suggested that NRH is metabolically handled by adenosine kinase (AK) and NMNAT in a sequential order to generate NADH, which can then be equilibrated to NAD^+^ through redox reactions [30,46]. NRH is also used by NRH:quinone oxidoreductase 2 (NQO2) as the electron donor to detoxify quinones, leading to the formation of endogenous NR [47]. 

## 3. Synthesis of NR

### 3.1. Biosynthesis of NR

The study of the biosynthesis of NR has been rather scarce. In yeast, NR can be produced via phosphatase-mediated NMN dephosphorylation [48]. The deletion of *nrt1*, an NR transporter, in a genetically altered yeast strain has resulted in increased secretion of NR [49], suggesting a possible biosynthetic approach for this vitamin. Endogenous NR biosynthesis has also been observed in mammalian cells [50]. It was further demonstrated that the dephosphorylation of NMN and NaMN by cytosolic 5′-nucleotidases (5′-NTs) led to the formation of NR and NAR in vitro.

### 3.2. Chemical Synthesis of NR 

**Figure 2:**
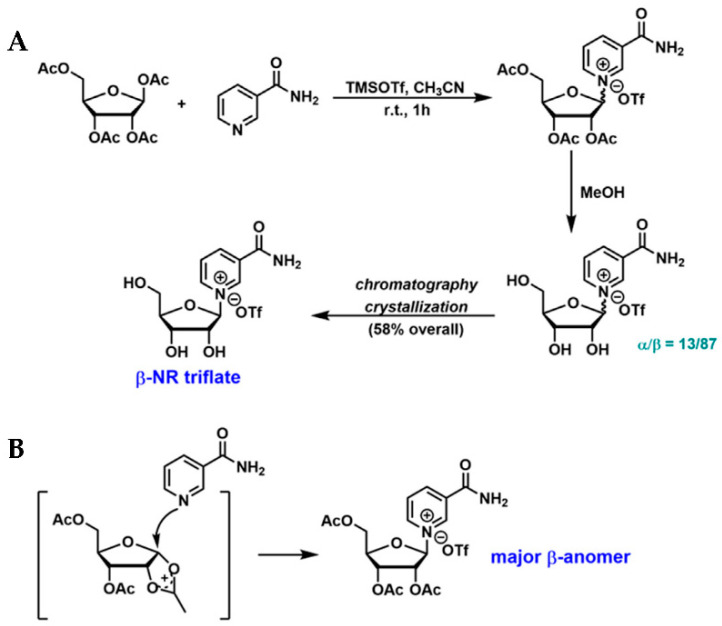
NR synthesis: glycosylation of NAM and acetylated-*D*-ribofuranose. (**A**) Synthetic scheme of β-NR triflate developed by Kirihata et al. [53]; (**B**) Neighboring group participation leads to the formation of β-NR as the major anomer.

The *N*-glycosidic bond in NR is considered the “weakest link” of the molecule. The presence of this labile chemical moiety poses a significant challenge in the chemical synthesis and modification of NR. The initial synthetic effort was focused on the coupling of NAM with peracylated-*D*-ribose, leading to the formation of a mixture of both α- and β-isomers with variable ratios [51,52]. A TMSOTf-mediated coupling reaction between NAM and commercially available tetra-*O*-acetyl-β-*D*-ribofuranose was later reported (Figure 2A) [53]. The glycosylation reaction was conducted in acetonitrile at room temperature, resulting in the formation of triacetylated NR. The subsequent methanolysis led to a mixture of both anomers in a 13:87 ratio (α:β), which was further purified by chromatography on activated charcoal and crystallization to afford the desired β-NR triflate in 58% overall yield [53]. The stereoselectivity of the glycosylation can be explained by “neighboring group participation”, as illustrated in Figure 2B [54]. 

**Figure 3:**
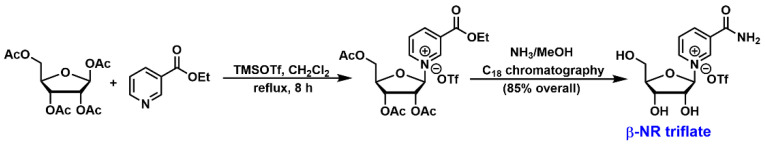
Synthetic scheme of β-NR triflate developed by Sauve et al. [39,55].

A simple two-step procedure was also developed to synthesize NR in a stereoselective manner [39,55]. Tetra-*O*-acetyl-β-*D*-ribofuranose and ethyl nicotinate in the presence of a stoichiometric amount of TMSOTf were refluxed in CH_2_Cl_2_ for 8 h (Figure 3). NMR results indicated the formation of only the β-anomer, suggesting that the coupling reaction went through an acyloxonium ion intermediate similar to the one shown in Figure 2B. The resulting ethyl nicotinate 2′,3′,5′-tri-*O*-acetylriboside triflate was then treated with ammonia in methanol for the simultaneous deprotection of acetyl groups and the conversion of ester to amide. The crude product was purified by C18 column chromatography to afford β-NR triflate in 85% overall yield. It is important to note that NR triflate is not a pharmaceutically acceptable form. Ion exchange with saturated sodium chloride solution provided NR chloride salt [56], which is commonly used as a dietary supplement. Other chemical syntheses of NR and its analogs have also been reported. Please refer to a wonderful review article on this topic if interested [57].

### 3.3. Chemo-Enzymatic Synthesis of NR

**Figure 4:**
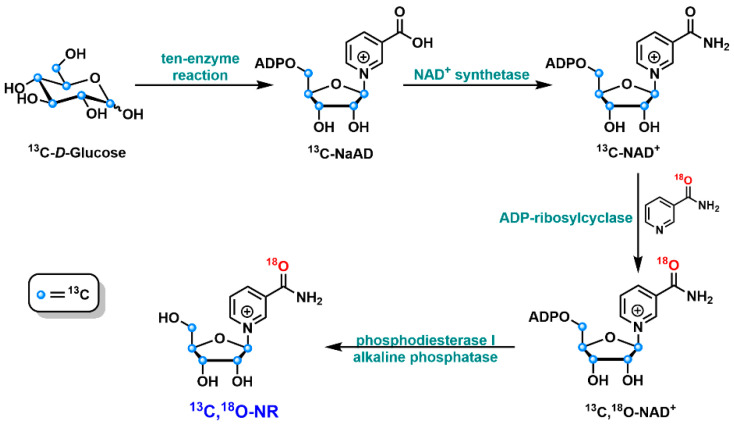
Schematic representation of the chemo-enzymatic synthesis of ^13^C, ^18^O-NR.

Accessing NR and its derivatives has also been explored using a chemo-enzymatic approach [58]. It started with a one-pot ten-enzyme coupled reaction to convert ^13^C-labeled glucose to ^13^C-labeled NaAD, which can then be transformed to NAD^+^ via NAD^+^ synthetase-catalyzed amidation reaction (Figure 4). The ^13^C-labeled NAD^+^ was then treated with chemically synthesized ^18^O-NAM in the presence of ADP-ribosylcyclase. This enzyme-mediated “base exchange” reaction allowed the formation of NAD^+^ with ^13^C labels in the ribose moiety and ^18^O label in NAM. The subsequent degradations of this NAD^+^ isotopomer by phosphodiesterase and alkaline phosphatase generated ^13^C, ^18^O-labeled NR in good yield. A similar method was applied to the formation of ^14^C-labeled NR [58]. These NR isotopomers have been used for the investigation of NR metabolism in the cellular setting. 

## 4. NR in Health and Diseases

As a potent NAD^+^ precursor, NR has profound implications for human health and diseases [59,60]. Many inflammation-related conditions—such as Alzheimer’s disease (AD), sclerosis, and fibrosis—are known to deplete NAD^+^ contents, aggravate cellular injury, and upregulate proinflammatory cytokines [19,61]. NR-mediated restoration of intracellular NAD^+^ pool has been shown to stimulate sirtuin activity; improve mitochondrial biogenesis and function; and provide benefits in health span and life span extension [17,62,63,64]. The role NR plays in neuroinflammation, fibrosis, and aging is discussed below and summarized in Table 1. It should be noted that despite the promising results in animal models, the therapeutic benefits of NR in human trials have been modest. This is partially due to the poor metabolic stability of this molecule. NR can be degraded to NAM in circulation, presumably by purine nucleoside phosphorylase (PNP) [14,65]. This degradation may compromise the clinical efficacy of NR. 

### 4.1. Neuroinflammation

Neuroinflammation is considered one of the common pathophysiological mechanisms of neurodegeneration [86]. Cytokine activation, pathogen-associated molecular patterns (PAMPs), or damage-associated molecular patterns (DAMPs) can lead to the formation and activation of NOD-like receptor protein 3 (NLRP3) inflammasome [87]. NLRP3 inflammasome activation subsequently upregulates caspase-1-mediated release of proinflammatory cytokines and promotes pyroptosis [87,88]. A declined NAD^+^ level has been identified as a distinct feature of neuroinflammation [66,67,89]. Therefore, the repletion of cellular NAD^+^ contents using NR may ameliorate neuroinflammation through the downregulation of inflammation-related pathways. Administration of NR in DNA repair-deficient AD mice improved cognitive functions and reduced neuropathological hallmarks of AD, presumably through the elevation of neuronal NAD^+^ levels and the subsequent stimulation of SIRT3 and SIRT6 activity [67]. In another transgenic AD mouse model, the increase in brain NAD^+^ via NR treatment downregulated NLRP3 inflammasome and proinflammatory cytokines and decreased the activation of neuronal immune cells, partially in a cGAS-STING-dependent manner [66]. AD-like alterations—such as an accumulation of Aβ aggregates and phosphorylated tau—can be triggered by high-fat-diet-induced brain insulin resistance [90]. In a type 2 diabetic mouse model, NR supplementation decreased neuroinflammation and amyloidogenesis with improved cognitive function [90].

Gulf War Illness (GWI), which occurs predominantly in veterans of the Gulf War [91], is characterized by impaired cognitive function, difficulties with memory, chronic fatigue, and pain. In a GWI mouse model, increased expression of proinflammatory cytokines IL-1β, IL-6, and interferon-gamma (IFN-γ) have been detected in the brains [68], along with decreased brain NAD^+^ and Sirt1. NR treatment not only restored NAD^+^ levels but also stimulated Sirt1 and Sirt3 activity for improved mitochondrial biogenesis and reduced neuroinflammation [68]. 

Amyotrophic lateral sclerosis (ALS) is a devastating neurodegenerative disease characterized by progressive loss of motor neurons [92]. NAD^+^ has been shown to exhibit remarkable neuroprotective properties in cultured neurons [93]. Indeed, the repletion of NAD^+^ using NR was shown to delay neurodegeneration, reduce neuroinflammation markers, and alter muscle metabolism in an ALS mouse model [69]. Furthermore, NAMPT, one of the NAD^+^ biosynthetic enzymes, was upregulated in ALS patients, suggesting an inherent regulation mechanism for the neurons [69]. 

### 4.2. Fibrosis

Liver fibrosis is a condition defined by the activation of hepatic stellar cells (HSCs) in response to DAMPs and the over-deposition of extracellular matrix proteins [94]. It leads to chronic inflammation and hepatocellular dysfunction [95]. An elevated serum level of alanine transaminase (ALT) is an indicator of hepatocellular injury [96]. In a CCl_4_-induced liver fibrosis mouse model, the oral administration of NR at 400 mg/kg significantly reduced serum ALT level and hepatocyte collagen deposition [75]. NR ameliorated liver fibrosis by restoring NAD^+^ contents, activating NAD^+^-dependent SIRT1 activity, and downregulating transcription coactivator p300. Subsequently, the TGF-β/Smads pathway-mediated HSC activation was inhibited, leading to reduced severity of liver fibrosis [75]. In other studies, the administration of NR did not reduce serum ALT levels significantly but decreased the levels of accumulated collagen and fibrotic markers [77,97]. In female C57BL/6J mice fed a high-fat diet (HF), NR supplementation at 400 mg/kg daily for 20 weeks did not improve live fibrosis remarkably. Rather, it improved the fibrosis in white adipose tissue in old (16 weeks) female mice [98].

In peripheral blood mononuclear cells (PBMCs), NAD^+^ augmentation by NR reduced the secretion of IL-6, a cytokine that is upregulated in patients with heart failure (HF) [78]. Additionally, the expression levels of IL-1β, IL-18, and NLRP3 inflammasome were also suppressed. Similar results were obtained in HF patients after oral NR administration [78]. Transforming growth factor-β1 (TGF-β1)-induced endothelial–mesenchymal transition (EndMT) contributes to the progression of cardiac fibrosis [99]. It has been suggested that TGF-β1-induced EndMT may regulate mitochondrial unfolded protein response (mtUPR) in endothelial cells [79]. NR treatment increased the expression of mtUPR, which was suppressed upon TGF-β1 exposure. Additionally, NR supplementation elevated the levels of prohibitin proteins, PHB and PHB2, the overexpression of which upregulated endothelial cell markers and the mtUPR marker and downregulated the fibroblast marker [79]. Moreover, transverse aortic constriction (TAC)-induced EndMT was also inhibited by NR in vivo, suggesting NR as a potential therapeutic for the treatment of cardiac fibrosis [79].

### 4.3. Aging

Aging is characterized by chronic inflammation and increased cell senescence. Together, these factors cause age-related disorders, such as cardiovascular diseases, osteoporosis, and diabetes mellitus [100,101]. Oral NR administration in aged participants has been shown to increase the levels of NAD^+^ and related metabolites in skeletal muscle and significantly reduce the levels of circulating inflammatory cytokines, such as IL-6, IL-5, IL-2, and tumor necrosis factor alpha (TNF-α) [80]. Amyloidosis, together with the loss of mitochondrial function, contributes to muscle aging in multiple species, such as *C. elegans*, mouse skeletal muscle, and human primary myotubes [81]. More importantly, NR-mediated NAD^+^ elevation restored muscle homeostasis and mitochondrial function and decreased muscle amyloid-like deposition in the same species [81]. In aged mice, elongation of villi and reduction in intestinal stem cell (ISC) number and function have been observed [82]. NR administration led to the inhibition of villi elongation and an increase in ISC population and function. Activation of the SIRT1/mTORC1 pathway upon NAD^+^ boosting has been suggested as the molecular mechanism of NR-mediated ISC rejuvenation [82]. 

Senescence is increasingly recognized as a key contributor to the aging process [102]. Ataxia telangiectasia (A-T), a rare premature aging disease, was characterized by senescence phenotypes [83]. At the cellular level, mitochondrial dysfunction and compromised mitophagy have been detected in A-T fibroblasts, and increased cytoplasmic dsDNA resulting from impaired DNA damage repair was observed in ataxia-telangiectasia-mutated (ATM)-deficient cells. NR treatment alleviated senescence phenotypes in cells via the inhibition of the stimulator of the interferon genes (STING) pathway, featuring enhanced mitophagy, restored mitochondrial function, and reduced cytoplasmic dsDNA [83].

Most of the studies on NR focus on its NAD^+^-increasing capability. A recent study documented its effect on NADH and NADPH levels in humans [84]. Acute NR administration increased erythrocytic NAD(P)H levels in young and old individuals. However, this treatment improved redox homeostasis and physical performance only in old individuals [84], highlighting the importance of further investigation on NR as an ergogenic supplement.

## 5. NR and COVID-19

The novel coronavirus SARS-CoV-2, the infective agent causing COVID-19, has caused a global pandemic and had a significant socio-economic impact. Novel targets and therapeutic interventions are highly sought after to combat deadly viruses. Multiple independent lines of evidence point to NAD^+^ metabolism as a potential target of intervention. Viral infections are known to cause cellular NAD^+^ depletion [103,104]. Indeed, declined levels of NMN, an NAD^+^ precursor, have been detected in the blood of COVID-19 patients [105]. Furthermore, the upregulations of PARP genes and NAD^+^ biosynthetic gene *nampt* were observed in SARS-CoV-2 infected individuals [23,106]. PARPs play key roles in antiviral immune response [107]. The induction of PARPs that are known to use NAD^+^ as the co-substrate to catalyze mono-ADP-ribosylation (MARylation) further decreased the cellular NAD^+^ contents. Moreover, the upregulation of NAMPT, the rate-limiting enzyme of the salvage pathway, can be viewed as a compensative mechanism in response to the increased demand for NAD^+^ [23]. Boosting intracellular NAD^+^ pool using precursors such as NR has been shown to block the replication of murine hepatitis virus (MHV) sensitive to MARylation PARP activity [23], lending support to the idea that restoration of NAD^+^ homeostasis may mitigate COVID-19 severity. 

NR is studied clinically in COVID-19 patients (Table 2). In one trial, the metabolic condition was investigated using a combination of NR and other metabolic cofactors, including *N-*acetylcysteine, *L*-carnitine tartrate, and serine together with hydroxychloroquine treatment (NCT04573153) [108]. The metabolic cofactors treated group demonstrated a significantly shortened recovery time with improved metabolic profiles [108]. In another trial, the dietary supplement of NR, Niagen, is evaluated for the improvement of recovery in patients suffering from Long-COVID (NCT04809974).

In addition to serving as an NAD^+^ booster, NR is also predicted to be a direct inhibitor of viral enzymes. SARS-CoV-2 RNA-dependent RNA polymerase (RdRp) regulates viral genome replication and gene transcription [109,110,111] and has been suggested as a potential therapeutic target for COVID-19. Nucleoside inhibitors (NIs)—such as Rivabirin [112] and Favipiravir—exhibited clinical efficacy against COVID-19 [113,114], presumably through the inhibition of RdRp activity [115]. NR, a structural mimic of the aforementioned NIs, has been proposed to have antiviral activity [116]. Molecular docking analysis and dynamic simulation studies suggested that NR may serve as a competitive inhibitor of SARS-CoV-2 RdRp, independent of its NAD^+^-elevating capability [116]. A recent docking study—together with target prediction, toxicity prediction, and ADME prediction—also hypothesized the clinical efficacy of NR in combating COVID-19 [117].

## 6. Conclusions

The initial discovery of NR-mediated lifespan extension in yeast without CR has ignited an intense interest in vitamin B3 for age-related studies [14]. NR supplementation has been increasingly recognized as an effective strategy to augment intracellular NAD^+^ concentrations to benefit human health. Chemical and enzymatic approaches have been developed to produce this rather labile molecule with good stereoselectivity, yield, and synthetic easiness. The biological function and therapeutic potential of NR have been heavily pursued in the last few years. This NAD^+^ precursor has been shown to prevent or alleviate multiple pathophysiological conditions in diverse model organisms. The clinical significance of NR is also investigated in several trials for metabolic disorders, aging, neurodegenerative diseases, and, most recently, COVID-19. Accumulating evidence unequivocally establish NR far ahead of other NAD^+^ precursors in improving human wellness. The future will reveal whether ample preclinical investigations can be translated into clinical applications.

## Figures and Tables

**Table 1:** Role of NR in health and different disease conditions.

Condition	Route of Administration	Mechanism of Action	Ref.
Neuroinflammation	Intracerebro ventricular	suppresses CD38-mediated neuroinflammation by increasing NAD^+^ levels and suppressing NF-κB in mice	[19]
	Oral (supplemented with drinking water) (12 mM) for 5 months	reduces NLRP3 inflammasome expression and proinflammatory cytokines in AD mouse model	[66]
	Oral (supplemented with drinking water) (12 mM) for 6 months	suppresses neuroinflammation in AD/Polβ mice by reducing the levels of proinflammatory cytokines IL-α, TNFα, MCP-1, IL-1β, MIP-1α and increasing the levels of anti-inflammatory cytokine IL-10	[67]
	Oral (supplemented with diet; 100 µg/kg daily) for 2 months	reduces inflammation in Gulf War Illness mice by increasing the deacetylation of NF-κB p65 subunit and PGC-1α	[68]
	Oral (supplemented with diet at 400 mg/kg); Oral (185 mg/kg)	decreases neuroinflammatory markers in amyotrophic lateral sclerosis (ALS) mice models	[69,70]
	Oral, via stomach gavage (400 mg/kg) for 6 weeks	reduces the level of amyloid-β precursor protein and inflammatory markers NLRP3, ASC, and caspase-1 in AD mice models	[71]
	Oral (400 mg/kg) for 4 weeks; Oral (supplemented with food 300 mg/kg) for 28 days	reversed the increased levels of TNFα in the hypothalamus of obese rats and cerebral small vessel disease mice	[72,73]
	100 µM for 24 h	suppressed endothelial inflammation by reducing ICAM1 and von Willebrand factor expression in IL-1β and TNFα-stimulated human aortic endothelial cells	[74]
Liver Fibrosis	Oral, via stomach gavage (400 mg/kg) for 8 weeks	reversed the development of CCl_4_-induced liver fibrosis in C57BL/6 mice by reducing TGF-β and serum ALT levels	[75]
	100 µM to 10 mM for 24 h	reduced the levels of proinflammatory cytokines TNFα and IL-6, and upregulated the levels of the anti-inflammatory molecule, adiponectin, in AML12 mouse hepatocytes	[76]
	Oral (400 mg/kg daily) for 20 weeks	Inhibits activation of HSCs by reducing the levels of fibrotic markers α-smooth muscle actin, collagen 1α1, and collagen 6α1	[77]
Heart failure and cardiac fibrosis	Oral (2 × 250–1500 mg daily) for 9 days	reduced the expression of proinflammatory IL-6 in PBMCs of individuals with Stage D heart failure	[78]
	Oral (400 mg/kg) for 6–8 weeks	improves the expression of prohibitin to suppress the progression of TGF-1β-induced endothelial-to-mesenchymal transition in cardiac fibrosis	[79]
	Oral (supplemented with diet at 400 mg/kg) for 4 weeks	improved mitochondrial function in heart failure with preserved ejection fraction mice by repleting NAD^+^ levels	[22]
Aging	Oral (1 g daily) for 21 days	reduces circulatory levels of inflammatory cytokines IL-2, IL-5, IL-6, TNFα and augments skeletal muscle NAD^+^ without altering its mitochondrial bioenergetics in humans	[80]
	Oral (400 mg/kg) for 8 weeks	reduces amyloid aggregation, improves mitochondrial membrane potential and function in mammalian cells	[81]
	Oral (supplemented with drinking water at 50 mg/kg) for 6 weeks	rejuvenates intestinal stem cells in aged mice by activating SIRT1 and mTORC1	[82]
	Oral (supplemented with drinking water at 12 mM) for 2 months	restores mitochondrial function and homeostasis in ataxia telangiectasia mice models	[83]
	Oral (500 mg)	improved physical performance and decreased oxidative stress in old individuals	[84]
	Oral (400 mg/kg) for 8 weeks	induces change in hematopoietic stem cells composition of aged mice towards a more youthful state by regulating the levels of mitophagy-promoting genes’ transcription	[85]

**Table 2:** Clinical trials of NR in COVID-19.

Treatment Regimen	Description	Type	Status	Clinical Trial
1 g of NR or placebo orally every morning for 14 days	to investigate whether NR supplementation can attenuate the severity of SARS-CoV-2 infections in elderly patients	randomized double-blinded case–control trial	Unknown	NCT04407390
250 mg NR capsules administered twice daily for 10 days	treatment with NR in COVID-19 patients for renal protection	prospective, double-blind, placebo-controlled clinical interventional trial	Active, not recruiting	NCT04818216
2000 mg NR in the form of capsules daily	to examine recovery in people with persistent cognitive and physical symptoms after COVID-19 illness	Double-blinded, randomized, parallel-group, placebo-controlled design	Recruiting	NCT04809974
hydroxychloroquine (standard therapy) + dietary supplement consisting of serine, *L*-carnitine tartrate, *N*-acetylcysteine, and NR	metabolic cofactor supplementation and hydroxychloroquine combination in COVID-19 patients	parallel-group, randomized, and open-label study	Recruiting	NCT04573153

## Data Availability

Not applicable.
